# Overview of Physical and Pharmacological Therapy in Enhancing Bone Regeneration Formation During Distraction Osteogenesis

**DOI:** 10.3389/fcell.2022.837430

**Published:** 2022-04-28

**Authors:** Ze Liu, Qi Liu, Hongbin Guo, Jieyu Liang, Yi Zhang

**Affiliations:** ^1^ Department of Orthopaedics, Xiangya Hospital, Central South University, Changsha, China; ^2^ National Clinical Research Center for Geriatric Disorders, Xiangya Hospital, Central South University, Changsha, China

**Keywords:** distraction osteogenesis, physical therapy, pharmacological therapy, bone healing, regeneration

## Abstract

Distraction osteogenesis (DO) is a kind of bone regeneration technology. The principle is to incise the cortical bone and apply continuous and stable distraction force to the fractured end of the cortical bone, thereby promoting the proliferation of osteoblastic cells in the tension microenvironment and stimulating new bone formation. However, the long consolidation course of DO presumably lead to several complications such as infection, fracture, scar formation, delayed union and malunion. Therefore, it is of clinical significance to reduce the long treatment duration. The current treatment strategy to promote osteogenesis in DO includes gene, growth factor, stem-cell, physical and pharmacological therapies. Among these methods, pharmacological and physical therapies are considered as safe, economical, convenience and effective. Recently, several physical and pharmacological therapies have been demonstrated with a decent ability to enhance bone regeneration during DO. In this review, we have comprehensively summarized the latest evidence for physical (Photonic, Waves, Gas, Mechanical, Electrical and Electromagnetic stimulation) and pharmacological (Bisphosphonates, Hormone, Metal compounds, Biologics, Chinese medicine, etc) therapies in DO. These evidences will bring novel and significant information for the bone healing during DO in the future.

## 1 Introduction

Ilizarov was the first one who utilized distraction osteogenesis (DO) on a canine model in the 1950s in Kurgan, Russia (Malkova and Borzunov, 2021). It attracted the attention of global orthopedic doctors. Currently, the Ilizarov technique is successfully used in many orthopedic reconstructive surgical procedures, such as deformities, pseudoarthrosis and osteomyelitis, congenital and developmental limb length discrepancy. Generally speaking, DO includes three clinical stages: distraction period, latency period, and consolidation period. The distraction period refers to the time required for stretching at a certain speed and frequency to reach the designed distraction gap. The latency period means the time from the placement of the tractor to the beginning of the retraction, which is generally 5–7 days. The length of the distraction period depends on the pre-operatively designed stretch gap. For example, if the planned traction is 20 mm, the distraction period is 20 days. The consolidation period represents the time from the completion of bone segment retraction to the removal of the distractor (Fujio et al., 2018).

**FIGURE 1:**
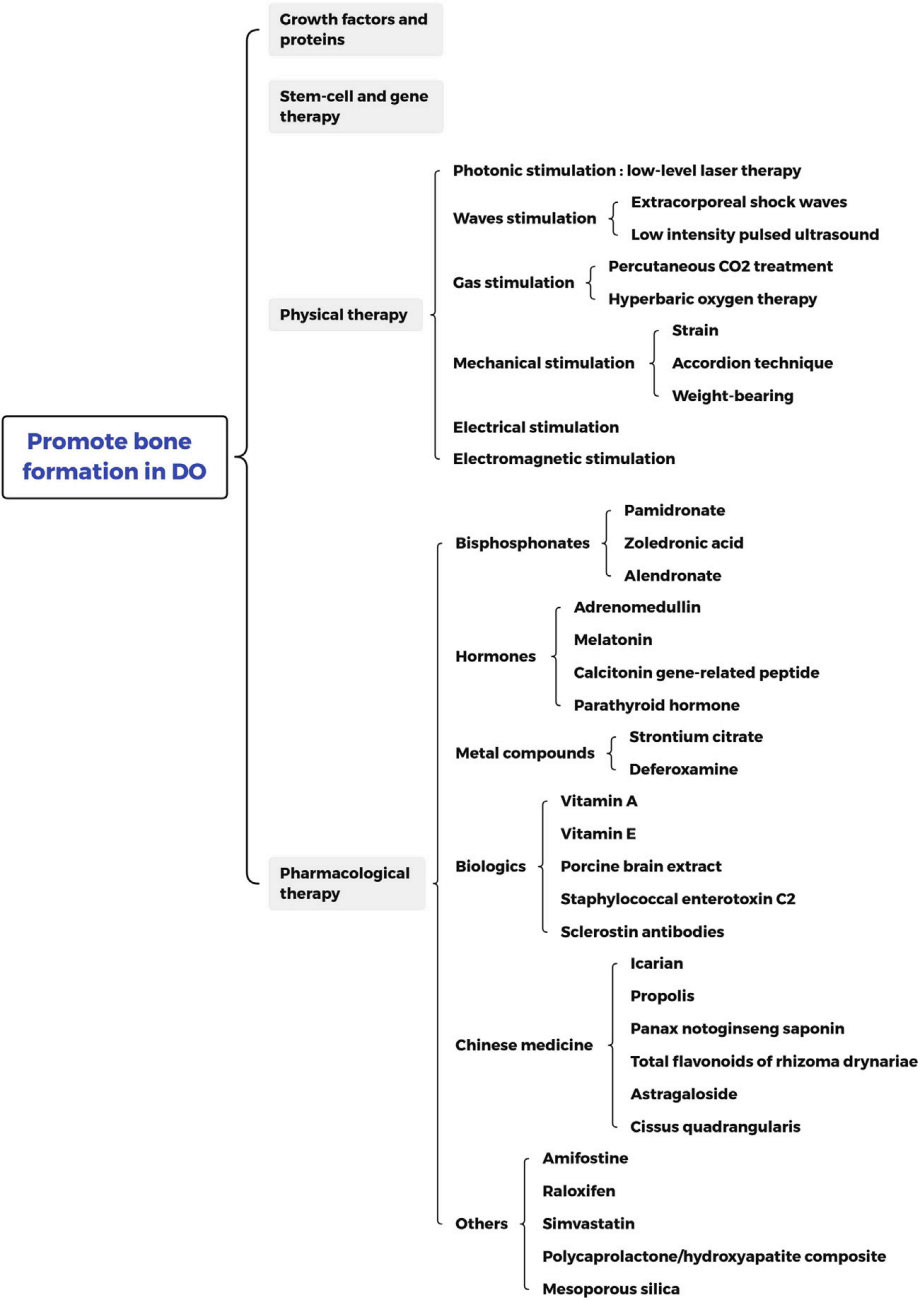
Overview of current therapy in enhancing bone regeneration formation during distraction osteogenesis.

**FIGURE 2:**
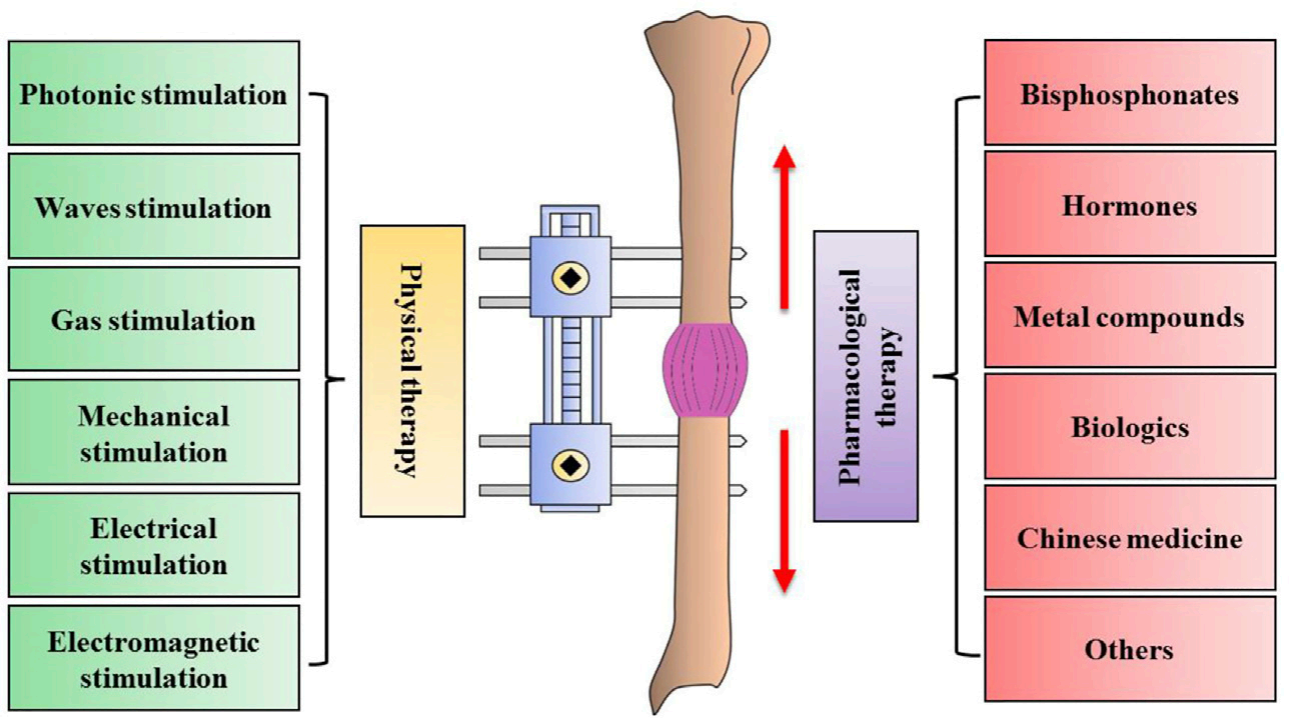
The detailed information for physical and pharmacological therapy in enhancing bone regeneration formation during distraction osteogenesis.

Although Ilizarov technology is relatively mature now, it still has some shortcomings. The long consolidation time for optimal novel bone tissue formation can induce several complications, such as refracture, pin loosening, nonunion of the distracted segments, and a psychosocial burden on patients (Dimitriou et al., 2011). Currently, growth factors, gene and stem-cell therapies have been utilized to accelerate the long consolidation period (Figure 1), However, the issues about safety, high cost, short biologic half-life, poor delivery methods, ectopic bone formation, selection of optimal dose and timing cannot be ignored. Physiological and pharmacological therapies are popular methods for its portability, non-invasiveness, and economical benefits, which has the advantages of easy access and controllability. Recently, great progress has been made in physical and pharmacological therapies for DO. Here, we have comprehensively summarized the latest evidence for physical (Photonic, Waves, Gas, Mechanical Electrical and Electromagnetic stimulation) and pharmacological (Bisphosphonates, Hormone, Metal compounds, Biologics, Chinese medicine and Others) therapies in bone healing during DO (Figure 2). These evidences will bring novel and significant information for the bone healing during DO in the future.

### 2 Physical Therapy

#### 2.1 Photonic Stimulation

Low-level laser therapy (LLLT), also known as photobiomodulation (PBM), is an equivocal cellular communication way to result in mitochondrial ATP production, cell signaling, and growth factor synthesis (Amaroli et al., 2019). To the best of our knowledge, LLLT could analgesia, reduce edema and speed up the tissue repair process (Yasukawa et al., 2007). Miloro et al. found that LLL (low-level laser) advanced bone trabeculation and ossification in histological, thereby accelerated the process of consolidation phase. It makes it possible to remove device earlier and avoids morbidity related to prolonged device retention (Miloro et al., 2007; Kreisner et al., 2010). The values of Crystallinity and Ca-to-P ratios that detected in the DO sites are strong evidence of bone maturity. Hubler et al. noticed that LLLT did not affect biological systems in homeostasis and had a positive effect on the chemical composition of newly formed bone (Hübler et al., 2010). Freddo et al. utilized Nanoindentation tests to evaluate bone quality, and found that ideal moment for laser application was consolidation period. The newly formed bone presents an increase in hardness and elastic modulus during this period, and the bone consolidation period is preferably at least 3 weeks (to avoid relapse) (Freddo et al., 2012). There are several complications that can arise in distraction process, such as bone defect formation and ulcers resulted from the components of distractor (Saulačić et al., 2007). LLLT can be safely utilized as a supportive treatment method to shorten the treatment period (especially the consolidation period) and increase osteogenic capacity for avoiding some complications (Kan et al., 2014).

Although the helium-neon laser was the first laser to be used, the gallium-aluminum-arsenite laser (GaAlAs) has become more prevailing due to its deep penetrating ability and significant biostimulative effects. Fazilat et al. employed a low-level GaAlAs (λ: 810 nm) laser in DO and demonstrated that LLLT promoted new bone formation in the early stages of the consolidation period, whereas it had no significant effect in later stages. It probably be the result of redox regulation when the cells exposed to laser light (Fazilat et al., 2014). Gurler et al. have investigated the effects of LLLT in DO in rabbit mandibles. They indicated that LLLT group presented more osteoblastic activity, angiogenesis and less cartilage formation than control groups (Gurler and Gursoy, 2018). Taha et al. concluded that LLL created a number of environmental conditions that could increase the osteoblast and osteoclast activity, enhance collagen synthesis, accelerate the healing of bone defects, stimulate the formation of bone callus and production of the bone matrix. Additionally, LLL had a significant biostimulatory effect on differentiation of mesenchymal cells into osteoblasts rather than chondroblasts (Taha et al., 2018).

Several clinical studies should also be noted. Abd-Elaal et al. recruited ten patients (seven females and three males) undergoing mandibular DO on both sides. They found that the use of LLLT on DO could increase the quality and quantity of bone, shorten the consolidation period, so that the distractor can be removed early and reduced morbidity and relapse. Those results are similar to Miloro et al.’ s (Abd-Elaal et al., 2015). Above all, laser therapy is a potentially effective method to promote bone healing in DO. However, further experiments should be employed to investigate the application of LLLT on the DO of long bones (limbs).

### 2.2 Waves Stimulation

#### 2.2.1 Extracorporeal Shock Waves

Extracorporeal shock wave therapy (ESWT) is a kind of sound wave with mechanical characteristics that generates energy through the extreme compression of the medium caused by vibration and high-speed movement (Opara et al., 2021). In the past decades, the functions of ESWT in the treatment of multiple disorders has been gradually realized (non-union of long bone fracture, pain relief, tissue regeneration, neuroprotection, chondroprotection and calcified tendinopathy) (Simplicio et al., 2020). Wang et al. found that the shockwave group presented significantly better bone strength in rabbit model, more cortical bone formed, higher number of neo-vessels and higher expression of osteogenic growth markers (VEGF, PCNA, eNOS and BMP-2). (Wang et al., 2008).

For the purpose of evaluating the effects of ESW on callus formation during bone elongation, Narasaki et al. applied ESWs on distracted calluses of rabbit tibias. At the twenty fourth weeks postoperatively, the formation of cortical bone in the ESW group was more obvious than that in the control group. Applying shock waves to scattered calluses leads to increased bone mineral density (BMD) and active bone formation. These results demonstrated the clinical potential of ESW in promoting callus maturation (Narasaki et al., 2003). Lai et al. also revealed an up-regulation of the expressions of VEGF, PCNA and BMP2 in ESWT group. This indicated that the optimal dosage of ESWT can enhance mandibular angiogenesis and bone regeneration, thereby accelerating bone consolidation. As mitogens or chemo-attractive modulators, the BMP2 and VEGF presumably can promote proliferation, maturation and migration in the distraction gap, as well as increase vessel formation, which cause granular tissue to transform into bone tissue (Lai et al., 2010). Moreover, different ESWT’s treatment protocols on DO were tested by Onger et al. Compare the double 500- and 1,000- shot doses of ESWT with control group. The results showed that all the different application types of ESWT had positive effects on DO. In each group, the most effective dose was found to be ESWT 1000 *2. They suggested that this dose can be used to shorten the consolidation process (Onger et al., 2017). However, Bereket et al. studied the effects of two different single doses of ESWT (500 and 1,000) on the consolidation period of DO in the rabbit mandible by using radiological, stereological and immunohistochemical methods. It demonstrated a statistically significant alternation in BMD and new capillary volume measurements. Apart from that, the highest values were obtained in the ESWT1000 group. Therefore, they considered that a total of 1,000 impulses ESWT might induce the growth factors to enhance new bone regeneration (Bereket et al., 2018). Overall, further studies are still needed to testify the optimal dose. Moreover, ESWT’s positive contribution to bone quality and mineralization might help prevent potential complications that may occur during long-term DO treatment. However, further experimental and clinical studies are still needed to confirm such findings.

#### 2.2.2 Low Intensity Pulsed Ultrasound

Low-intensity pulsed ultrasound (LIPUS) is an oscillating longitudinal pressure wave whose frequency cannot be detected by human auditory systems. It has a low intensity to exert therapeutic effects (< 3 W/cm ^2^) (Harrison and Alt, 2021). As an adjuvant therapy, LIPUS is gradually used in the clinical treatment of fresh fractures, delayed fracture union and nonunion. In addition to this, LIPUS is a strategy with non-invasive and convenient, which has very minimal side effects. The therapeutic effects of LIPUS are mostly attributed to its predominantly cavitation, acoustic radiation force and acoustic streaming. Cavitation generally occurs in liquid materials and is believed to be related to the changes in cell membrane permeability and cell activation. Acoustic radiation force is able to influence the cardiovascular systems, and acoustic streaming is in charge of the diffusion rate and changes of protein synthesis, cellular secretion (Xu et al., 2021). The acoustic stimulations activate cell surface integrins, and then in turn activate various mechanical transduction pathways including FAK, ERK, PI3K, and Akt *in vitro*. Then, the production of prostaglandin E2 and cyclooxygenase 2 is activated to stimulate further downstream osteogenic, angiogenic and chondrogenic cytokines. Interestingly, it indicates a different enhancements between in animal and clinical studies (Zhang et al., 2017).

Recently, LIPUS has been widely investigated in both animal experimental and clinical research. Ding et al. investigated the effect of LIPUS on new bone formation during mandible DO in dogs, and concluded that LIPUS could accelerate bone formation during mandibular DO and increase BMD (Ding et al., 2009). Lü et al. demonstrated that LIPUS was a biophysical stimulation that might accelerate the maturation of regenerated bone in rabbit tibia elongation model (Lü et al., 2009). In addition, Xie et al. further suggested that LIPUS was effective in the early stage of DO, such as 2 weeks after the distraction period as well as the distraction period (Xie et al., 2011).

Clinically, Dudda et al.’s randomized controlled trial enrolled thirty-six patients who underwent DO. Their results indicated that therapeutic application of LIPUS during callus distraction constituted an effective adjuvant treatment, which had a positive effect on healing time without negative effects (Dudda et al., 2011). Salem et al.’ proved that LIPUS treatment was effective to enhance callus maturation in DO (the overall daily increase in callus density in the LIPUS group was 33% higher than that in the control group radiographically). It promotes callus consolidation and might have a positive effect on the favorable callus shape (Song et al., 2019). With the assistance of LIPUS, the healing time can be significantly shortened (fixator time of LIPUS group was shortened by 95 days), which may prevent the development of pseudarthrosis, delayed union and some other fixator-related complications, such as joint contractures and pin tract infection (Salem and Schmelz, 2014).

Kocyigit et al. utilized dual energy x-ray absorptiometry (DEXA) to compare the effects of LIPUS and LLLT on the BMD of formed bone during DO. They ultimately reported that LLLT and LIPUS were both safe and noninvasive procedures that could improve the outcome of DO treatment (Kocyigit et al., 2012). Andrade Gomes do Nascimento et al. further indicated that the influences of ultrasound and low-intensity laser irradiation on BMD were statistically significant, and a greater BMD recuperation was obtained in the mandibular side by ultrasound application (Andrade Gomes do Nascimento et al., 2013). Apart from that, Medeiros et al. found that the combination of ultrasound and laser treatment presented the greatest effects (Medeiros et al., 2015).

In order to deliver realistic benefits to humans, further clinical researches are still needed to address various issues, such as wavelength for lasers, dose, duration, and application frequency etc. for LIPUS in terms of their effects on soft and hard tissue.

### 2.3 Ga Stimulation

#### 2.3.1 Percutaneous CO ₂ Treatment

Sakai et al. designed an original system for transcutaneous application of carbon dioxide using pure carbon dioxide gas, hydrogel and a plastic adaptor (Sakai et al., 2011). CO₂ treatment increased blood flow and the expression of VEGF at the bone defect sites, resulting in bone defect healing and accelerated endochondral ossification in rat femoral defect model (Kuroiwa et al., 2019). Therefore, Kumabe et al. adopted DO in a rabbit model to testify the cutaneous application of CO₂ in bone generation in DO. They showed that the gene expression of BMP-2, VEGF, HIF-1α were higher in the CO₂ group than that in control group. These results indicated that CO₂ therapy could accelerate angiogenesis, bone formation, and promote oxygen supply at the sites of skeletal generation. In conclusion, the transcutaneous application of CO₂ may be possible to shorten healing time in patients with DO (Kumabe et al., 2020). In summary, the percutaneous application of CO₂ seems to be a new method for shortening the consolidation period in patients with DO. However, more translational studies is still necessary before its clinical application.

#### 2.3.2 Hyperbaric Oxygen Therapy

The use of hyperbaric oxygen therapy (HBOT) decreases the ability of neutrophils to adhere to the vessel walls, thereby reducing endothelial damage and allowing vasoconstriction of vessels in regions having a normal oxygen concentration. It restores collagen production and fibroblast growth, and the storage of adenosine-triphosphate (ATP) in cell membranes. It exerts a great impact on the reduction of edema in tissues, limiting some forms in immune response, stimulating capillary proliferation, osteoclasts activity (Olex-Zarychta, 2020). Clark et al. evaluated the effect of HBOT on bone regeneration during distraction in both irradiated and non-irradiated rabbit mandibles. They showed adjunctive HBOT was beneficial in the healing of non-irradiated bone (Clark et al., 2006). Kudoh A. et al. demonstrated that bone mineral density (Salgado et al., 2009), blood flow, and the newly formed bone area were greatly higher in the HBOT group than that in the control group. These results suggested that HBOT could be helpful for early removal of the distraction device in DO (Kudoh, 2008).

In addition, HBOT can overcome the adverse effects of distraction to the skeletal muscle, promote recovery in post-ischemic muscle and preserve the contraction capacity of the muscles by modulating the antioxidant enzyme activity (Atesalp et al., 2009). Inokuchi et al. analyzed the effect of HBOT on newly formed bone in distracted areas, which accelerated ossification and vascularization of newly formed bone histologically and radiologically (Inokuchi et al., 2010). Mutlu et al. concluded that HBOT could be useful for increasing the quality and the quantity of bone, and reducing the maturation time to shorten the consolidation period of DO (Mutlu et al., 2012). Moreover, Kaynar et al. indicated that HBOT promoted the adaptation of distracted muscle by increasing antioxidants and fibers while decreasing inflammation (Kaynar et al., 2014). Taken together, clinical studies are still stagnant nowadays. Further clinical studies are urgently needed to eliminate the latency period and shorten the consolidation time by using HBOT.

### 2.4 Mechanical Stimulation

#### 2.4.1 Strain

Mechanical stimulation plays a critical role in DO for decades. However, the mechanism of mechanical stimulation to bone regeneration during DO remain elusive. Song et al. suggested that the mechanical stimulus induced high expression of Fak and activated Fak-Mapk pathway which played a significant role in maintaining osteogenic effect during DO (Song et al., 2018).

According to Qi et al.’ studies, mechanical strain can increase ALP activity, promote MSC (mesenchymal stem cells) proliferation and up-regulate expression of one specific osteoblast marker (ALP) as well as two osteogenic transcription factors (Cbfa1 and Ets-1). Therefore, mechanical strain might be served as a stimulator to induce differentiation of MSC into osteoblasts, which is key to bone formation in DO (Qi et al., 2008). Static stretching is the main type of mechanical stimulation utilized in DO. A combined treatment of BMP-2 with stretching load stimulation greatly increased alkaline phosphatase (ALP) activity and up-regulated the expression of osteogenic markers (ALP, osteopontin, osteocalcin, type I collagen, osterix, cbfa1, and dlx5). These results showed that specific levels of static stretching strength can increase cell proliferation and successfully stimulated the osteoblast differentiation of C2C12 cells in conjunction with BMP-2 stimulation, indicating a synergistic interaction between cytokine signaling and mechanical strain (Kim et al., 2009). Similarly, Yang et al. suggested that static strain may promote the migration capability of BMMSCs (bone marrow mesenchymal stem cell) via p38/MMP-2 signaling (Yang et al., 2017). Li et al. used a homemade device applying 10% uniaxial static strain on osteoblasts, they showed that the cell growth direction of the osteoblasts became orderly and the cytoskeleton microfilaments were rearranged. Moreover, uniaxial static strain improved the osteogenesis, proliferation, and angiogenesis of osteoblasts, respectively (Li et al., 2020). Actually, the effect of strain on DO is still at the preclinical stage. Further clinical trials are rather needed.

#### 2.4.2 Accordion Technique

Accordion maneuver is a cycle of distraction-compression that can be used to accelerate bone regeneration. Compressive forces can cause osteogenesis, fibrogenesis and intramembranous bone formation, while distraction forces may lead to chondrogenesis and endochondral bone formation. According to relevant literature, adding outside compressive forces during the lengthening process is beneficial for bone formation, and compression after over-distraction, or dynamization of the fixator are mentioned as the accordion maneuver (Makhdom et al., 2015). Makhdom et al. suggested that the accordion technique was noninvasive and safe approach to promote bone formation, which avoided more invasive surgical procedures and prevented poor callus formation in limb elongation (Makhdom et al., 2015). Shen et al. designed an experiment to investigate the effect, right timing, and mechanism of “accordion” technique on bone regeneration in rat DO model. They drawn the conclusion that the “accordion” technique was beneficial for new callus formation in distraction area. The middle phase of the consolidation period was the best choice to apply the maneuver to accelerate new bone formation in rat DO model (Shen and Ye, 2018). With regard to the mechanism, immunohistochemical and histomorphological analyses confirmed a greater degree of osteogenesis and angiogenesis corresponding to increased serum levels of VEGF and HIF-1α in Group 2 (the accordion technique used in the middle phase of bone consolidation). The accordion technique was effective in promoting bone consolidation via activating HIF-1α/VEGF during DO (Xu et al., 2018). In addition, more in-depth clinical trials are a prerequisite for promoting clinical applications.

#### 2.4.3 Weight-Bearing

It was reported that more new bone formed in the distraction site of the weight-bearing animals. The expression of BMP 1/4 and the messages for osteocalcin and collagen I were more abundant in tissue of the weight-bearing animals. This suggested that early regenerating tissue was able to respond to loading, and weight-bearing seemed to stimulate intramembranous ossification. These findings supported the opinion of early weight-bearing after limb elongation (Radomisli et al., 2001). More specifically, micro-tomographic imaging study confirmed that weight bearing seemed to increase the number and length of newly formed vessels, but did not influence vessel caliber (Moore et al., 2003). The significant increase in the expression of TGF-β1 during the distraction phase in the weight-bearing group were attributed to both the additive effects of weight-bearing stimulation and continuous distraction (Leung et al., 2004). The mechanical environment is a primary factor in the success of DO. Therefore, its clinical application should be encouraged in further study. A retrospective chart review conducted by Bafor et al. They suggested that pixel value ratio can be used to evaluate the healing of regenerated bone by comparison with adjacent bone density, and can be a practical choice for routine follow-up as it was a readily available method to determine when it was favorable to start weight-bearing (Bafor et al., 2020).

### 2.5 Electrical Stimulation

Electrical stimulation (ES) and electromagnetic stimulation have been applied to many aspects, such as electrocardiogram, brain electricity, etc. Under ES, voltage-gated calcium channels on cell membranes are opened, and the influx of Ca (Fujio et al., 2018)+ results an increase of intracellular Ca (Fujio et al., 2018)+, then activates calmodulin (CaM) and calcineurin. As a calcineurin phosphate factor, the nuclear factor of activated cells (NF-AT) is transported to the nucleus and cooperates with other transcription factors to induce gene transcription, for examples, the transcription of bone morphogenetic proteins (BMPs), transforming growth factor β (TGF-β), and some other growth factors. They are responsible for proteins synthesis, cell metabolism and extracellular matrix (ECM) production (Zheng et al., 2020).

Hagiwara et al. investigated the effect of ES on mandibular DO. They demonstrated that new bone was successfully formed by 10 mA ES directly. It was possible that ES promoted new bone formation during DO when the electric current level was adequate. They also found that ES promoted both the proliferation of osteoblasts and the differentiation of mesenchymal cells into osteoblasts in the distraction gap. Therefore, direct ES may shorten the overall duration of elongation (Hagiwara and Bell, 2000). For the purpose of establishing the optimal period to apply an electric current during the distraction process. El-Hakim et al. performed the experiments in twelve healthy adult goats. They indicated that when this stimulus applied to the distraction zone during distraction or consolidation periods, direct current ES had a synergism effect on mandibular distraction (El-Hakim et al., 2004). However, the kind of electricity commonly used in daily life is alternating current. In Kawamoto et al.’ study, alternating current (AC) ES been known as easier and less invasive, and simultaneously accelerated bone formation (AC stimulation changed the polarity and caused oxidation of the anode). The obtained results revealed that AC stimulation accelerated the maturation of lengthened callus radiologically, electro-physiologically and histologically, which shorten the time course of callus formation (Kawamoto et al., 2005). Moreover, according to Peña-Martínez et al.’ study, a significant difference in the bone density and callus formation between experimental group and control group was obtained, which demonstrated the effectiveness of AC stimulation during bone elongation can speed up the consolidation process in animal model (Peña-Martínez et al., 2017). Despite the widespread use of electromagnetic stimulation in orthopaedics, there is no definitive clinical data support its use in DO or bone lengthening. Both researchers and device manufacturers should better understand the potential role of electromagnetic stimulation in DO through using important patient outcomes and well-designed clinical trials.

### 2.6 Electromagnetic Stimulation

Pulsed electromagnetic fields (PEMFs), a constant change of the magnetic field amplitude over time, are low frequency magnetic fields with a specific waveform and amplitude. The pulsed magnetic field produces a secondary electric field in the exposed tissue, which is similar to the one naturally generated during the conversion of mechanical energy into electrical energy (Varani et al., 2021). PEMFs are currently widely applied to promote reparative osteogenesis (Massari et al., 2019). Fredericks et al. conducted a randomized controlled trial for 16 days after distraction, the PEMF group attained biomechanical strength essentially equal to intact bone, whereas control group (no exposure to PEMF) failed even after 23 days post-distraction. Therefore, short daily PEMF exposures promoted consolidation of regenerate bone in the tibial distraction model (Fredericks et al., 2003). Taylor et al. found that the application of PEMF in the early phase was more effective, since it was reported that applying the same PEMF signal in the distraction phase and consolidation phase was beneficial to the rabbit model. Further study is needed to determine the optimal timing of PEMF stimulation during DO (Taylor et al., 2006).

With regard to the clinical trials, Luna Gonzalez et al. enrolled thirty patients to undergo bilateral bone elongation of the tibia, femur or humerus. At 10th day after osteotomy, PEMF stimulation was initiated on one side for 8 hours/day. Stimulated distraction areas exhibited a higher callus density and earlier callus formation (compared with non-stimulated sites). External fixation can be removed 1 month earlier than that in PEMF stimulated bones. They showed that the utilization of PEMF during limb elongation shortened the timing of external fixation (Luna Gonzalez et al., 2005). Overall, the clinical evidence is rather limited, which need to be expanded in the future.

## 3 Pharmacological Therapy

### 3.1 Bisphosphonates

#### 3.1.1 Pamidronate

Bisphosphonates are synthetic analogs of inorganic pyrophosphate, which have the property of a phosphorus-carbon-phosphorus bond with a strong affinity for bone mineral. Their metabolism in blood cycle are rapid, and they are easily combined with bone minerals and accumulated within bones selectively. Bisphosphonates hinder bone resorption by directly acting on the function of osteoclasts, and also hinder the osteoblastic recruitment of osteoclasts. The actions and potency of bisphosphonates vary depending on the configuration of a side chain, and pamidronate has an amino side chain (Diel et al., 2007).

Little et al. examined the effect of a single dose of 3 mg/kg of the bisphosphonate, pamidronate (Novartis) on BMD in immature rabbits DO model. They found that Pamidronate had a significantly positive effect: it reduced the disuse osteoporosis that was usually associated with the use of an external fixator to lengthen and it increased the number and density of the regenerate bone. Before clinical application, it is necessary to check the mechanical properties of the bone after treatment with pamidronate, the possible toxicological effects and refinement of the dosage regimen (Little et al., 2001a).

#### 3.1.2 Zoledronic Acid

Zoledronic acid (ZA) is an effective inhibitor of bone resorption because it directly effects on osteoclasts. The bisphosphonate is released and internalized by the osteoclasts, interfering with survival, formation, and function of osteoclast. ZA is 18 times more effective than pamidronate (even in small doses) (Little et al., 2001b). Baiomy et al. designed an experiment to figure out the effects of topical zoledronic acid compared with alendronate on bone formation in DO. A higher statistically significant difference was found in ZA group (more mature and accelerated bone) (Baiomy et al., 2014). Pampu et al. assessed the effect of systemic application of ZA on mineralization of newly formed bone, and their results showed that ZA can dramatically promote the new bone formation, which may potentially accelerate the consolidation period (Pampu et al., 2008). Although systemic administration of bisphosphonates (BPs) has been proved effective in accelerating bone formation in DO, several complications, such as osteonecrosis of the jaw, nephrocalcinosis and gastrointestinal disorders should not be ignored. Clinical symptoms of these complications may take several years to appear. The local application of BPs in DO might help eliminate most complications caused by systemic administration, and accelerate bone tissue formation. Therefore, Dundar et al. claimed that the local application of ZA could be safe and advantageous during the DO consolidation period in a clinical condition (Dundar et al., 2017). Saghieh et al. found that bisphosphonate-treated rabbits had a stronger regeneration: There was no disuse osteopenia in their lengthened tibia. This treatment might shorten the course of the external fixator and effectively prevent fragility fractures (Saghieh et al., 2010). In Akbulut et al.’ experimental study, they confirmed the effects of a single dose of 0.1 mg/kg or 0.2 mg/kg systemically administered zoledronic acid, when compared with the controls, may be an effective strategy for promoting new bone maturation in rat femurs with DO (Akbulut et al., 2021). The strong and mature bones lay a good experimental foundation for future clinical research.

#### 3.1.3 Alendronate

Alendronate is known as one of the most potent BPs in inhibiting bone resorption. The detailed mechanism of this inhibition is unclear. However, the inhibition of osteoclast development from precursor cells, the down-regulation of matrix metalloproteinases, reduction of osteoclast activity, stimulation of osteoclast inhibitory factor and an increase in osteoclast apoptosis are considered to be involved. In Tekin et al. experimental study, the results shown that alendronate administration during the distraction period might be useful for accelerating new bone formation in the distraction site in rabbit mandibles (Tekin et al., 2008). Intravenous injection of bisphosphonate has been proved to cause myalgia, nausea, renal toxicity and fever. Therefore, the ideal approach is to minimize the dose of bisphosphonate to avoid those detrimental effects and maximize the impact on the highly activated bone resorption during DO. Abbaspour et al. used an osmotic pump to examine the effects of bisphosphonate’ continuous injection on bone volume, architectural and mechanical properties of DO. They suggested that alendronate infused in this way greatly avoided the osteopenia that critically started early in the consolidation period (Abbaspour et al., 2009). Kucuk et al. compared systemic and local alendronate groups in their animal experiment, and the results showed that systemic alendronate is more effective than local alendronate in accelerating new bone formation (Küçük et al., 2011). In Alp et al.’ utilized a rate of 1 mm/day lengthening and locally injected low-dose alendronate in rabbit mandibles, which provided the optimal new bone formation. This suggested that bisphosphonates might be beneficial in clinical practice (Alp et al., 2017).

There was a clinical trial conducted by Patrick Kiely et al. (Kiely et al., 2007) Seven patients who used an Ilizarov device for limb lengthening were found to have insufficient regeneration. With informed consent, patients received intravenous bisphosphonate treatment. Six of the patients’ fixators were removed without requirement for other intervention, which demonstrated a sustained and rapid improvement in local BMD. While the left one patient did not respond and subsequently received percutaneous osteogenic protein 1 (BMP 7) and bone marrow injection. Therefore, these minimally invasive approaches may reduce the need for surgical reintervention. Nevertheless, the detrimental effects of bisphosphonates include bleeding, ulceration, nausea, inflammation, osteonecrosis of the jaw, and abdominal pain in the upper gastrointestinal tract should be taken into seriously consideration when using these kinds of drugs in humans (Kos et al., 2010).

### 3.2 Hormone

#### 3.2.1 Adrenomedullin

Adrenomedullin 2 (ADM2) is an endogenous biologically active peptide belonging to the family of calcitonin gene-related peptide. It has a variety of biological activities related to the attenuation of ischemic-hypoxic damage and the inhibition of inflammation. Both osteogenic differentiation and the pro-angiogenic potential of BMMSCs promote bone regeneration during DO. Wang et al. suggested that ADM2 could improve the osteogenic differentiation and pro-angiogenic potential of BMSCs by inhibiting NF-κB and activating AKT signaling through the accumulation and activation of β-catenin. This indicated that ADM2 (as a novel bioactive factor) was capable of decreasing the consolidation phase during DO treatment (Wang et al., 2021a). In the process of DO, bone regeneration is a complex physiological process which is regulated by various factors, and multiple metabolic disorders impair bone regeneration during DO. Diabetes mellitus (DM)-induced impairment of bone regeneration, a relatively common condition characterized by a prolonged mineralization phase, leads to discomfort and complications. A previous study shown that systemic ADM2 levels of diabetic rats were significantly decreased, indicating that there is a relationship between low ADM2 levels and DM-related metabolic disorders (Li et al., 2013). Currently, Wang et al.’ study demonstrated that ADM2 partly reversed AGE-induced M1/M2 imbalance through the IκBα/PPARγ/NF-κB signaling pathway and simultaneously restored the bone formation potential of BMSCs that is damaged by AGE, revealing ADM2 as a novel method to promote bone regeneration under diabetic conditions during DO (Wang et al., 2021b). However, more clinical trials are needed to confirm the drug’s safety in human use.

#### 3.2.2 Melatonin

The bone tissue remodeling is organized by the activity of multiple hormones (melatonin, parathyroid, oestradiol, and growth hormones). Among them, melatonin is a particular interesting one for its osteoblastic activity. Melatonin is an indolamine hormone derived from tryptophan, which is mainly released by the pineal gland. Moreover, it could also be found in many tissues, such as the intestinal mucosa and retina. Since the free oxygen radicals can destroy the bone tissue regeneration process, and melatonin is known to be an important scavenger of free oxygen radical. It can also hinder bone tissue resorption because it suppresses osteoclast production. In addition to this, melatonin can increase osteoblast proliferation and differentiation *in vitro* (Dundar et al., 2016). The morphometrical and immunohistochemical outcomes in Acikan et al.’ study uncovered a melatonin-induced dose-dependent increase for new bone regeneration in DO (Acikan et al., 2018). In general, there are lacking of relevant experiments to demonstrate the association between Melatonin and DO.

#### 3.2.3 Calcitonin Gene-Related Peptide

To the best of our knowledge, the effects of calcitonin are mainly to inhibit bone resorption by osteoclasts and increase the bone mineralization rate (Sen et al., 2006). Calcitonin gene-related peptide (CGRP) belongs to the calcitonin superfamily of peptides consisting of amylin, calcitonin, adrenomedullin 2, adrenomedullin, and calcitonin-receptor-stimulating peptide. Calcitonin is primarily produced by thyroid C cells whilst CGRP is produced and stored in the nervous system. Jia et al. found that CGRP application increased new bone formation, possibly through promoting BMMSC migration and differentiation in mandibular distraction osteogenesis (MDO) rats (Jia et al., 2019a). According to reports, CGRP promoted angiogenesis by affecting endothelial progenitor cells (EPCs) in ischemic limbs and injured wounds (Mi et al., 2021). The therapeutic effect of CGRP is closely related to the increased EPCs population at the distraction gap, which may be derived from BMSCs via activating PI3K/AKT signaling pathway. As far as we know, relevant experiments are still insufficient at this stage, and further well-designed clinical trials are rather needed.

#### 3.2.4 Parathyroid Hormone

As the main hormonal regulator of calcium-phosphate homeostasis, PTH has a dual effect on skeletal metabolism. Although continuous high-dose PTH will accelerate bone resorption, intermittent treatment of low-dose PTH stimulates the activities of osteoblastic and osteoclastic, which has a greater effect on bone formation than resorption. Ali et al. used microcomputed tomography to confirm the effect of intermittent PTH(1–34) treatment on DO in a rat model, and obtained a desirable results. Meanwhile, they found out that intermittent PTH(1–34) treatment might be beneficial to accelerate the consolidation period (Ali et al., 2012). Besides, according to Tsiridis et al. the osteogenesis seen with PTH and OP-1 treatment might bring on magnificently accelerated and enhanced regain of mechanical capacity of the injured metaphyseal bone in a dependable model of metaphyseal bone healing (Tsiridis et al., 2007). In DO process, faster distraction rates might decrease the patient immobility and treatment course. However, it has been shown to lead to a delayed osteogenesis and fibrous tissue formation. By using a rapid mandibular DO model, Yamashita et al. demonstrated that PTH effectively compensated for the adverse effect on new bone formation caused by a rapid distraction rate (Yamashita and McCauley, 2019). The clinical significance of the findings is that the administration of intermittent PTH might be helpful for patients with DO and poor regenerate quality, especially for patients whose osteogenic potentials are impaired by osteoporosis, aging, and post-oncologic irradiation (Kang et al., 2017). The optimal timing of PTH (1–34) administration in DO is during the consolidation phase. Inada et al. complemented that this period was primarily characterized by intramembranous ossification (Inada et al., 2022). Teriparatide (rhPTH) is the active fragment of endogenous human PTH. A growing number of delayed or nonunion of fractures emerge. Osagie-Clouard et al. used external fixator to establish a delayed union model and testified the favorable effect of localized MSC injections on fracture healing combined with low- or high-dose teriparatide, with efficacy dependent on PTH dose (Osagie-Clouard et al., 2021). Wagner et al. enrolled sixteen patients to undergo a clinical study, and concluded that teriparatide treatment during the consolidation period might double the mineralization rate of the regeneration. Therefore, teriparatide has the potential to shorten the DO consolidation period clinically (Wagner et al., 2019).

### 3.3 Metal Compound

#### 3.3.1 Strontium Citrate

Strontium, an element that has been frequently used to treat osteoporosis, has an affinity for bone. It has been reported to hinder bone resorption and stimulate new bone formation in animal models. The current study found that strontium citrate effectively accelerated the formation of new bone in the MDO rabbit model (Taylor et al., 2017). Therefore, oral intake of strontium citrate may be a potential strategy to promote bone regeneration in the distraction site. An obvious advantage is that strontium is a safe over-the-counter medication that can be administered orally without another procedure. Prior to human use, it is necessary to further test different doses of strontium citrate with varied distraction and consolidation periods to determine the most beneficial plan for maximal bone regeneration.

#### 3.3.2 Deferoxamine

Deferoxamine (DFO) is an angiogenic activator triggering the HIF-1α pathway through localized iron depletion. Recent tissue engineering researches have revealed the use of angiogenesis and vasodilation as mechanisms to enhance bone regeneration in long bone murine models. Based on these findings, Donneys et al. contended that increasing vascular density through localized DFO injection provided an useful method to accelerate bone regeneration without significantly affecting bone quality or strength (Donneys et al., 2013). More specifically, Donneys et al. found that the angiogenic effect of deferoxamine would improve bone regeneration by promoting the quality and quantity of bone and the number of osteogenic cells (Donneys et al., 2012). Except for irritation at the injection site, they did not observe any clinically adverse events. Therefore, they concluded that for the purposes of enhancing the bone regeneration abilities and augmenting the vascular response mandibular DO, this would be a relatively affordable and safe strategy (Farberg et al., 2014). The clinical application of deferoxamine is still at the exploratory moment, and more clinical evidence are rather indispensable.

### 3.4 Biologics

#### 3.4.1 Vitamin A

As an active metabolite of dietary vitamin A, all-trans retinoic acid (ATRA) is involved in the regulation of various biological processes, such as cell proliferation, differentiation and migration. Moreover, Bi et al. indicated that ATRA promoted angiogenetic capacity, facilitated bone healing and enhanced the expression of angiogenic genes (ANG-2, ANG-4, and VEGF) in DO (Bi et al., 2013). Weng et al. found genes related to osteogenesis, including BMP2, Runx2, and ALP were all significantly up-regulated following ATRA-treatment. They concluded that ATRA enhanced bone formation and consolidation and promoted osteogenic differentiation of rBMSCs (rat bone marrow-derived mesenchymal stem cells) during DO in a rat model. These findings suggested that ATRA (off-the-shelf and very cost-effective) might be a promising medication for accelerating bone consolidation during DO treatment in patients (Weng et al., 2019). However, these experimental evidences are far from sufficient. Further clinical studies should be considered.

#### 3.4.2 Vitamin E

During the bone formation, the activation of macrophages, neutrophils, and mast cells generates oxygen-derived free radicals. These free radicals increase the production of osteoclast, leading to insufficient bone generation. It has been proved that the application of antioxidants is beneficial to inhibit the negative effects of oxygen free radicals in the process of new bone formation. Besides, vitamin E is known for its recognized antioxidative properties. Alpha-tocopherol, a Vitamin E derivative has been found to act as a biological antioxidant preventing lipid peroxidation caused by free radicals derived from free oxygen. The findings of the Akcay et al.’ study suggested that systemic alpha-tocopherol administration during DO may stimulate new bone formation and increase the number of vessels, osteoblasts and osteoclasts in rabbits by quantitative and radiologic bone morphological evaluations (Akçay et al., 2019). Accelerated bone healing might reduce the detrimental effects of extremely prolonged distraction and consolidation time, which can enhance the patient comfort. However, since vitamin E is commonly consumed in daily life, the comparison of these two administration needs to be addressed by further experiments.

#### 3.4.3 Porcine Brain Extract

The factors from neural tissues have been found to be effective mitogens for mesoderm-derived cells, especially some ectoderm-derived cells and vascular endothelial cells. Because of their ability to stimulate proliferation of fibroblasts, these growth factors have been named brain fibroblast growth factors (FGFs). The porcine brain accumulates various growth factors, including FGF, has been traditionally regarded as food supplements to improve brain function. It is well known that the FGFs derived from the porcine brain extract (PBE) can regulate proliferation and differentiation of MSCs into mature osteoblasts. Osteogenesis-related genes including Runx2, ALP, OCN, Col1α, and BMP2, which were dramatically upregulated following PBE treatment. Moreover, the FGFs in PBE are mitogens and homeostatic factors for vascular endothelial cells. FGFs play a significant role in tissue repair, angiogenesis and vascular permeability. Xu et al. reported that PBE promoted osteogenic differentiation of rBMSCs, and their local treatment accelerated bone formation and consolidation in a rat DO model (Xu et al., 2017a). These findings indicate that PBE might be a promising bio-source to be utilized to promote new bone regeneration (due to it is extremely cost-effective and easily available). Since these extracts may be restricted to their safety concerns, future experiments should be employed to explore their side effects.

#### 3.4.4 Staphylococcal Enterotoxin C2

Staphylococcal enterotoxin C2 (SEC2) has been found to inhibit osteoclastogenesis of MSC (Fu et al., 2014). Xu et al. examined the changes of osteogenic marker genes in the SEC2 treated rBMSCs, and revealed that Runx2, ALP, OPN, OCN, and Osx were all upregulated by SEC2 treatment (Xu et al., 2017b). Their results proved that SEC2 augmented osteogenic differentiation of rBMSCs, and its local repeated application at low dose obviously accelerated bone formation and consolidation during DO in a rat model. Therefore, SEC2 may be a promising treatment to promote bone formation and consolidation period in patients undergoing DO treatment. Since the current evidence is rather limited, and more experimental research is still required in the future.

#### 3.4.5 Sclerostin Antibodies

Sclerostin is a glycoprotein that is only secreted by osteocytes. It interacts with the LRP5/6 receptor, thereby restraining the intracellular Wnt signaling pathway, resulting in a decrease in bone formation activity. Antagonizing sclerostin can promote bone formation during implant fixation and fracture healing, and may be beneficial to treat osteogenesis imperfecta and low bone mass in the context of estrogen-deficient osteoporosis. Makhdom et al. claimed that the systemic delivery of sclerostin antibodies (Scl-Ab) led to augment of bone regeneration during the distraction period in DO (Makhdom et al., 2014). McDonald et al.’ demonstrated that Scl-Ab treatment increased bone formation, promoted regeneration with higher strength and bone volume. Moreover, the optimal effects of Scl-Ab treatment can be presented in the latter stages of DO (McDonald et al., 2018). Anti-Scl-Ab has been shown to be more effective than bisphosphonates and teriparatide in promoting bone formation by Alzahrani et al. (Alzahrani et al., 2018) In addition, lower doses of Scl-Ab therapy with less frequent dosing could provide patients a more cost-effective tolerable and safer treatment experience.

### 3.5 Traditional Chinese Medicine

Traditional Chinese Medicine (TCM) represents a largely number of untapped resources of modern medicine. Taking the success of the antimalarial artemisinin as an example, the understanding and application of TCM-derived herbs has increased rapidly in recent years. In this section, the relationship between TCM and DO will be fully discussed to provide a novel direction for future research (Wang et al., 2018).

#### 3.5.1 Icariin

Wei et al. conducted a RCT (randomized controlled trial) to assess the effect of icariin on bone formation during mandibular distraction of rabbits. The experimental group was given icariin (2.5 mg/kg/day), and the control group was given a placebo. At the fourth week, the experimental group had higher trabecular number, greater volumes of new bone, and less trabecular separation than the controls. Therefore, they drawn the conclusion that oral administration of icariin can accelerate bone formation during mandibular DO and may be a potent agent to shorten the duration of DO (Wei et al., 2011). Similarly, more clinical trials are needed to verify its efficacy in humans.

#### 3.5.2 Propolis

Similar research was performed by Bereket et al. At the fourth week after surgery, the P200 group (200 mg/kg/d of propolis given orally) showed accelerated bone formation. The data of this study suggested that systemically administered propolis accelerated bone formation. Propolis treatment is practical, noninvasive, easily administered and cheap. However, allergies to bee products (bee venom) are potential challenges. This method may reduce the consolidation duration and avoid complications related to a prolonged distraction period. Therefore, it is recommended to use propolis to obtain a satisfactory clinical outcome. Further researches are needed to determine the optimal dose and the effects of different doses (Bereket et al., 2014). Cafffeic acid phenethyl ester (CAPE) is one of the components of propolis, which is produced by bees. Multiple studies have proved that it has antibacterial, antiseptic, antioxidant, antimutagenic, anti-inflammatory and immunomodulator effects. CAPE also can promote the maturation of new bone in DO. These effects are probably on account of the reducing effect on bone resorption by restraining NF-κB and free oxygen radicals (Erdem et al., 2014). To compare the effects of CAPE with other traditional Chinese medicine, further experimental and human studies should be performed.

#### 3.5.3 Panax Notoginseng Saponin

Panax notoginseng saponin (PNS) as a traditional Chinese medicine, frequently used to treat a series of diseases. Herein, rabbit BMSCs and a model of mandibular DO were used to explore the capabilities of PNS treatment to affect osteogenic differentiation by Liu et al. For *in vivo* experiments, treated BMSCs were locally injected into the DO site, with PNS being injected into treated rabbits every other day throughout the experiment. The quality of the regenerative process was assessed via energy dispersive spectroscopy (EDS), scanning electron microscopy (SEM), hematoxylin and eosin (H&E) staining and X-ray imaging. These analyses revealed that PNS was capable of enhancing mandibular generation and BMSC osteogenesis, upregulating osteogenesis-related genes at the mRNA levels through the modulation of the Smad/TGF-beta1 pathway. To sum up, these results proved the PNS as a potential and cost-effective therapeutic tool (Liu et al., 2021). However, a single experiment lacks credibility, and further in-depth experiments are essential to confirm the relationship between PNS with DO.

#### 3.5.4 Total Flavonoids of Rhizoma Drynariae

The purpose of Sun et al.’ study was to assess the effect of TFRD (Total flavonoids of rhizoma drynariae) on bone formation and angiogenesis in LTDs (large tibial defects) in DO. The 12th week after treatment, according to Micro-CT and X-ray, TFRD groups can significantly improve new bone mineralization and promote the formation of epiphyses compared with model group (with only DO). Besides, angiographic imaging indicated that total flavonoids of TFRD was capable of promoting angiogenesis in the defect site. Consistently, TFRD dramatically increased the levels of SMAD1, SMAD4, BMP-2, VEGF, RUNX-2 and OSX in LTD rats based on Real-Time PCR and ELISA. Therefore, they claimed that TFRD accelerated bone formation in LTD via activation of BMP-Smad signaling pathway, which provided a potent agent for repairing bone defects in DO surgeries (Sun et al., 2021). Therefore, the issues of dosage should be considered in the future.

#### 3.5.5 Astragaloside

Astragaloside IV (AS-IV), a glycoside of cycloartane-type triterpene derived from the Chinese herb *Astragalus* membranaceus, possesses various biological activities, including attenuating ischemic-hypoxic injury and stimulating angiogenesis. In Wang et al.’ study, they found that AS-IV promoted bone regeneration during DO, by enhancing preosteoclast-induced angiogenesis and osteogenesis simultaneously, partially through GSK-3beta/AKT/beta-catenin signaling. These findings indicated that AS-IV might serve as a promising bioactive molecule for enhancing the coupling of angiogenesis and osteogenesis, and implied that GSK-3beta/AKT/beta-catenin signaling might be a potential therapeutic target for patients during DO treatment (Wang et al., 2021c). However, these conjectures should be further confirmed in experiments.

#### 3.5.6 Cissus Quadrangularis

Cissus quadrangularis (CQ) is a kind of medicinal plant that has been frequently utilized as a traditional medicine to heal injured bones, ligaments and tendons. It could induce metabolism and enhance minerals absorption by osteoblasts. Altaweel et al. conducted a randomized clinical trial and found that Cissus quadrangularis could not only promote new bone formation, but also bone density to withstand the biomechanical requirements (Altaweel et al., 2021). The small sample size is a limitation of this study, and more experimental and clinical studies to evaluate the potentiating effect of CQ in DO are necessary.

### 3.6 Others

#### 3.6.1 Amifostine and Raloxifen

Amifostine (AMF) is a radioprotectant. Tchanque-Fossuo et al. suggested that AMF was capable of improving the mineralization metrics of irradiated bone after DO. The novel application of AMF prophylaxis could be beneficial for optimization of DO (Tchanque-Fossuo et al., 2018). In addition to this, Leiblein et al. supplemented that antiosteoporotic drugs like raloxifen might be useful as a stimulator of new bone regeneration in DO (Leiblein et al., 2020).

#### 3.6.2 Simvastatin

Statins (including simvastatin) are utilized to lower cholesterol levels, and are competitive inhibitors of 3-hydroxy-3-methylglutaryl-coenzyme A reductase and its upstream metabolites (such as mevalonate). Besides, it has been revealed that statins promote bone formation and angiogenesis both *in vitro* and *in vivo*, which is related to increased expression of the BMP-2 and VEGF gene in bone cells respectively (Garrett et al., 2001). Indeed, the effect of topical simvastatin on bone formation is carrier- and dose-dependent (Hao et al., 2018). Undoubtedly, further clinical trials to testify its safety are indispensable.

#### 3.6.3 Polycaprolactone/Hydroxyapatite Composite

The application of biomaterials for bone tissue engineering is a potent therapeutic strategy and significant progresses have been made during the last 2 decades. With excellent mechanical properties, remarkable biocompatibility and biodegradability polycaprolactone (PCL) is severed as one of the most attractive polymers for bone regeneration. However, wider application in bone tissue engineering of pure PCL has been limited by poor cell affinity, relatively low stiffness, and its hydrophobic nature. Therefore, combining bioactive inorganic particles with PCL polymer (such as hydroxyapatite (HA)) can enhance microsphere surface roughness, the osteo-conductive and mechanical properties of the microspheres. Wen et al. found that PCL/HA composite microspheres could promote osteogenic differentiation of rBMSCs through the accumulation of VEGF and the activation of BMP2 signaling pathway *in vitro* (Wen et al., 2020). Their results implied that local injection of PCL/HA composite microspheres may be a new strategy to enhance bone formation in patients experiencing DO treatment.

#### 3.6.4 Mesoporous Silica

Si ions are considered to have osteostimulative properties. Recent studies have demonstrated that nanoparticles can promote the differentiation of MSCs toward osteoblasts through various mechanisms (Shi et al., 2015). Therefore, it has been suggested that mesoporous silica nanoparticles could stimulate the osteogenic differentiation of MSCs by delivering silicon (Si) ions. Jia et al. synthesized mesoporous silica coated magnetic (Fe3O4) nanoparticles (M-MSNs) and evaluated its ability to stimulate bone regeneration in a rat DO model. The M-MSNs showed excellent biocompatibility and noticeable capability to promote the osteogenic differentiation of MSCs through the canonical Wnt/β-catenin pathway *in vitro*. In addition to this, local injection of M-MSNs significantly stimulated bone regeneration *in vivo* (Jia et al., 2019b).

## 4 Discussion

DO is widely used in orthopedic surgery, including lengthening limbs, correcting deformed bones, and treating bone defects caused by infection, trauma, and tumors. DO largely makes up for the deficiencies of other techniques such as autologous bone grafting and allogeneic bone grafting. Although the clinical application effects of DO technology is favorable, there are still some problems to be solved in the process of distraction, including poor callus regeneration in the distraction gap and refracture after callus regeneration. The process of DO involves applying a controlled traction force to both ends of the bone defect to guide new bone formation through three phases: the distraction phase, latency phase, and consolidation phase. Despite the unique ability to fully induce new bone formation, this lengthy technique is limited by the prolonged duration of the consolidation phase and the increased risk of subsequent complications. Therefore, it is of great clinical significance to accelerate the formation and consolidation of callus, and shorten the external fixation time.

LLLT may improve bone density, accelerate healing, and appear to have anti-inflammatory and analgesic effects (Santinoni et al., 2017). In qualitative analysis, the results of most studies were positive. Although the LLLT group showed more new bone and osteoblasts during cell division, the improvement was not statistically significant (Freddo et al., 2016). Therefore, additional clinical trials should be conducted to confirm the effectiveness of LLLT in improving bone density. The animal experiments of ESWT have the effect of improving the bone mechanical properties and mineralization without obvious side effects. This method can be easily applied in an outpatient setting (no anesthesia required) with a short learning curve. Moreover, extracorporeal shock wave therapy is considered as a reliable treatment. Nevertheless, its effect in humans has not been fully elucidated. LIPUS has been widely used to promote the fracture healing process. However, its clinical value in DO remains questionable. Although LIPUS has many advantages, a formal and consistent LIPUS protocol is still needed.

Regarding the effect of gas stimulation on DO, animal experiments and clinical trials are extremely lacking in the lower limbs. Slow and continuous mechanical force can promote tissue metabolism, increase biosynthetic function, stimulate cell proliferation, and thus lead to tissue regeneration. Moreover, the type, magnitude, frequency, and duration of mechanical stimulation also affect osteoblast responses, which should be considered in future studies. On the other hand, electromagnetic stimulation for DO is less concerned than electrical stimulation. More importantly, the combination of electrical and electromagnetic stimulation in DO worth further investigation.

Bisphosphonates, zoledronic acid and alendronate are effective methods to accelerate bone regeneration in DO. Among these three pharmacological agents, zoledronic acid have been proved to be more preferable than the other two. However, the issue of dosage are required to be addressed in further clinical research. Moreover, adrenomedullin, calcitonin, calcitonin gene-related peptide, etc. are still at the preclinical stage (animal experiments). Therefore, further research should specify their molecular mechanism and physiological role of the potential therapeutic effect. Further understanding the role of these hormones, thereby facilitates hormone-related bio-therapeutics into the clinical setting. In addition, Mg, Zn, Cu, Mn and Co have been proved to exert favorable effects on bone regeneration (Li et al., 2021). Although the research on biologics is still in its infancy, it can be seen as a promising approach for its good biocompatibility. On the other hand, the importance of traditional Chinese medicine is gradually being recognized. However, unfortunately, the research on traditional Chinese medicine is still limited. Given that traditional Chinese medicine may be a potential clinical candidate for accelerating the consolidation period and shortening the treatment time, the mechanism behind should be specified by further study.

## 5 Conclusion

Currently, several strategies were considered to enhance bone regeneration formation during DO: gene, growth factor, stem-cell, physical and pharmacological therapy. Among them, physical and pharmacological therapy are safe, low cost, non-invasive, effective ones. In this review, we have comprehensively summarized the latest evidence for physical and pharmacological therapy in DO, which will bring novel and significant information for the bone healing during DO. Moreover, the combination of multiple methods might be considered as an effective measure for tissue reconstruction and bone healing in the future.
